# 

**Published:** 1871-10

**Authors:** 


					﻿VOL. XII.
OCTOBER, 1871.
NO. 10.
CHICAGO
MEDICAL EXAMINER
N. S. DAVIS, M. D., Editor.
AND
F. H. DAVIS, M. D., Assistant Editor.
WWiSMSl. $^.OO EEIl	IM EllIVKiMEW
ANN ARBOR:
COURIER STEAM PRINTING HOUSE.
1S7 1.
				

